# The Applicability of Biomonitoring Data for Perfluorooctanesulfonate to the Environmental Public Health Continuum

**DOI:** 10.1289/ehp.9060

**Published:** 2006-06-12

**Authors:** John L. Butenhoff, Geary W. Olsen, Andrea Pfahles-Hutchens

**Affiliations:** 1 3M Company, Medical Department, St. Paul, Minnesota, USA; 2 U.S. Environmental Protection Agency, Office of Prevention, Pesticides, and Toxic Substances, Washington, DC, USA

**Keywords:** biomonitoring, exposure assessment, perfluorooctanesulfonate, PFOS, public health paradigm

## Abstract

Perfluorooctanesulfonate and its salts (PFOS) are derived from perfluorooctanesulfonyl fluoride, the basic chemical building block for many sulfonyl-based fluorochemicals used as surfactants and for their repellent properties. PFOS is highly persistent in the environment and has a long serum elimination half-life in both animals and humans. PFOS has been detected globally in the environment and in blood serum in various populations throughout the world, with the majority of human sampling done in the United States and Japan. The mechanisms and pathways leading to the presence of PFOS in human blood are not well characterized but likely involve both direct exposures to PFOS or chemicals and materials that can degrade to PFOS, either in the environment or from industrial and commercial uses. In 2000 the 3M Company, a major manufacturer, announced a phaseout of PFOS-related materials. Animal studies indicate that PFOS is well absorbed orally and distributes mainly in blood serum and the liver. Several repeat-dose toxicology studies in animals consistently demonstrated that the liver is the primary target organ. In addition there is a steep dose response for mortality in sexually mature rats and primates as well as in neonatal rats and mice exposed *in utero*. Several biomonitoring research needs that have been identified on PFOS include additional data from general populations pertaining to other matrices besides blood; matched serum and urine samples from humans and research animals; and comparison of whole blood, serum, and plasma concentrations from the same individuals.

Perfluorooctanesulfonate and its salts (PFOS) are fully fluorinated organic molecules that were produced synthetically by electrochemical fluorination or from the degradation or metabolism of other fluorochemical products produced by electrochemical fluorination. PFOS and its precursors all belong to the larger class of fluorochemicals known as perfluoroalkyl substances and were derived from perfluorooctanesulfonyl fluoride (POSF), the basic chemical building block for many sulfonyl-based fluorochemicals. POSF is used primarily as an intermediate to synthesize numerous fluorochemicals, including PFOS.

3M Company produced POSF, PFOS, and POSF-related materials for over 40 years. These materials exhibit chemical and physical properties that can be substantially different from hydrogenated analogs, and perfluorinated portions of the molecules have extreme resistance to environmental and metabolic degradation. The strength of carbon–fluorine bonds contributes to the extreme stability and unique properties of PFOS. PFOS, as a surfactant, has high surface tension–reducing properties, lower water and oil solubility, and is a relatively strong organic acid. The commercial uses of PFOS included predominantly surface treatments for soil- and stain-resistant coating on fabrics, carpets, and leather, coatings on paper and packaging products for grease and oil resistance, including food contact papers, and performance chemicals uses such as fire-extinguishing foam concentrates, mining and oil surfactants, electroplating and etching bath surfactants, household additives, chemical intermediates, coatings and coating additives, carpet spot cleaners and insecticide raw materials (3M Company 2003). Total worldwide POSF production by 3M Company in 2000 was approximately 8 million pounds. However, on 16 May 2000, 3M Company announced that it would globally phase out the perfluorooctanyl chemistry used to produce certain repellents and surfactant products, which included the manufacture of PFOS and related compounds (3M Company 2000). 3M steadily reduced their production volume and discontinued the manufacture of most PFOS and POSF-based chemicals by 31 December 2002 (3M Company 2001). Manufacture of PFOS for certain uses for which no substitutes are available are continuing by non-U.S. producers [U.S. Environmental Protection Agency (U.S. EPA) 2002].

The purpose of this article is to provide a brief overview of the biomonitoring data for PFOS in the context of the public health paradigm as discussed in Albertini et al. (2006). We place emphasis on reviewing PFOS data related to those parts of the public health paradigm covering exposure, dose, and potential health outcomes in an effort to develop a view of the sufficiency of the PFOS biomonitoring data for use in public health.

## Serum PFOS Concentration as a Biomarker of Exposure

PFOS has been measured primarily in human blood serum (Harada et al. 2004, 2005a; Kannan et al. 2004; Kubwabo et al. 2004; Kuklenyik et al. 2004; Olsen et al. 2003b, 2004b, 2004c); however, data are also available on PFOS in human whole blood (Falandysz et al. 2006; Inoue et al. 2004a; Kannan et al. 2004; Masunaga et al. 2002; Yeung et al. 2006), plasma (Inoue et al. 2004b; Kannan et al. 2004; Olsen et al. 2005b), liver (Olsen et al. 2003c), cord blood (Inoue et al. 2004a), breast milk (Kuklenyik et al. 2004), and seminal plasma (Guruge et al. 2005). PFOS was measured in the liver and serum of cadavers (Olsen et al. 2003c). The average PFOS serum and liver data for each of 23 paired samples (serum and liver from the same individual) showed a good correlation for both male and female donors, and the mean liver to serum ratio was 1.3:1 [95% confidence interval (CI), 0.9:1–1.7:1). Mean PFOS levels for male and female donors were similar for both serum (male = 18.2 ng/mL; female = 17.2 ng/mL) and liver (male = 19.2 ng/g; female = 28.4 ng/g).

Quantitative analysis of PFOS from selected matrices is challenging and has been conducted using various extraction techniques and instrumentation methods (Ellefson and Reagen 2005; Martin et al. 2004). Matrix effects (Ellefson and Reagen 2005) and isomeric forms (Kärrman et al. 2005) should be taken into account. Extracted matrix calibration curves (i.e., not just water blanks) should be used to ensure the accuracy of laboratory analysis. Limits of quantitation are in the low nanogram per milliliter range; however, interlaboratory variability is significant, based on the results of the PERFORCE (PERFluorinated ORganic Chemicals in the European environment) interlaboratory study (PERFORCE 2005). In the PERFORCE study, 17 participating laboratories produced values for a spiked serum standard that varied with a relative standard deviation of 31.5%. For whole blood, 11 participating laboratories produced values that varied with a relative standard deviation of 56.1%. Liquid–liquid extraction techniques, or a variation thereof that involves ion pairing (Hansen et al. 2001), have been used for extraction of PFOS from biological matrices and have involved internal standards through the complete extraction. Chromatography and quantitation have typically been accomplished using high-performance liquid chromatography–mass spectrometry (HPLC-MS) in negative ion mode, with quantitation of the parent ion (LC-MS) or transition ions (HPLC-MS/MS). Solid-phase extraction techniques (Kuklenyik et al. 2004; Taniyasu et al. 2003) and column-switching methods (Inoue et al. 2004a, 2004b) are examples of recent improvements in the quantitation of PFOS. Mass-labeled internal standards such as ^18^O_2_-PFOS will result in improved quantitation.

Organically bound fluorine was reported in human blood in 1968 (Taves 1968) and before the development of analytical techniques for specific fluorochemical analytes, total organic fluorine was used to measure organic fluorine in human blood (3M Company 2003). In an attempt to speciate the organic fluorine in blood using ^19^F nuclear magnetic resonance, Taves et al. (1976) tentatively suggested the presence of perfluorooctanoate and also noted that branching or the presence of a sulfonate was a possible interpretation of their findings. As HPLC-MS methods were developed, “blank” human serum was found to contain PFOS in 1997. Subsequently, PFOS was found distributed widely in human serum and fish-eating wildlife serum and liver (Giesy and Kannan 2001; Hansen et al. 2001).

## Pharmacokinetics

The pharmacokinetic properties of PFOS are favorable for using serum PFOS concentration as a measure of internal dose. Good absorption, lack of known metabolism, distribution primarily in extracellular space, high serum protein binding (albumin and beta-lipoproteins), and poor elimination in all species studied combine to establish serum PFOS concentration as an integration of exposures from various sources. In addition, serum PFOS concentrations can be directly associated with effects in toxicology studies. PFOS serum concentrations can be used as a marker for total exposure in occupational medical surveillance studies.

Animal studies indicate that PFOS is well absorbed orally and distributes mainly in the serum and liver, with liver concentrations being potentially several times higher than serum concentrations (Johnson et al. 1979a; Seacat et al. 2002b, 2003). The volume of distribution at steady state, as measured in cynomolgus monkeys, is approximately 200 mL/kg, suggesting distribution primarily in extracellular space (Noker and Gorman 2003). PFOS is highly bound to albumin and has affinity for binding to β-lipoproteins (Jones et al. 2003; Kerstner-Wood et al. 2003) as well as albumin and liver fatty acid–binding protein (Luebker et al. 2002a). PFOS is poorly metabolized and excreted and undergoes extensive enterohepatic circulation (Johnson et al. 1984). The elimination half-life of PFOS is approximately 100 days in rats (Johnson et al. 1979b) and 100–200 days in cynomolgus monkeys (Noker and Gorman 2003; Seacat et al. 2002b) and appears to be approximately 5 years in human serum (Olsen et al. 2005a).

## Exposure Assessment

The mechanisms and pathways leading to the presence of PFOS in human blood are not well characterized but likely involve environmental exposure to PFOS or to precursor molecules and residual levels of PFOS or PFOS precursors in industrial and commercial products. Potential sources of human exposure to PFOS may have included manufacturing operations and waste streams of POSF-based fluorochemical products and the use or degradation of some final commercial and consumer products, including indirect food-contact applications (3M Company 2003). Other potential sources may include exposure to airborne PFOS, POSF, and *N*-alkyl-perfluorooctanesulfonamides (Martin et al. 2002; Sasaki et al. 2003; Stock et al. 2004), surface water (Boulanger et al. 2004; Saito et al. 2004; So et al. 2004), sediments and sludge (Higgins et al. 2005), and indoor air and dust (Shoeib et al. 2004, 2005; Strynar and Lindstrom 2005). PFOS has been identified in serum and tissue samples from both occupationally and nonoccupationally exposed human populations in various countries, in various species of wildlife in many parts of the world, and in surface waters and other environmental media in various countries (3M Company 2003; Giesy and Kannan 2001; Hansen et al. 2001, 2002; So et al. 2004). PFOS is highly persistent in the environment and has also been shown to bioconcentrate in fish and biomagnify to an extent in the food chain.

The only two countries in which multiple biomonitoring investigations of general population PFOS levels have been undertaken are the United States (Table 1) and Japan (Table 2). The total number of samples presented in these two countries represents approximately 90% of the individual analyses published in the literature. The similar distribution of averages (as well as similar ranges) from individual studies presented in Table 1 suggests that serum PFOS concentrations are relatively comparable across geographic regions and age groups in the United States. However, the most recent pooled serum data indicate modest differences by sex as well as ethnicity (Calafat et al. 2006). In addition, the range of serum concentrations in the U.S. populations indicates that some individuals, albeit a small number, may have had relatively higher exposure levels than the majority of individuals sampled. Average PFOS concentrations were lower among the Japanese populations presented in Table 2. In one Japanese study, Harada et al. (2004) observed sex-related differences in serum PFOS concentrations, with males approximately 2-fold higher than females. Furthermore, Harada et al. (2005a) reported higher concentrations among premenopausal than postmenopausal women in another study. These findings were not seen in general populations in the United States (Kannan et al. 2004; Kuklenyik et al. 2004; Olsen et al. 2003b). In the only published study of its kind, PFOS was measured in 15 pairs of maternal and cord blood (fetal) samples from Japan (Inoue et al. 2004a). PFOS concentrations in maternal samples ranged from 4.9 to 17.6 ng/mL, whereas those in fetal samples ranged from 1.6 to 5.3 ng/mL, with a high degree of correlation between pairs (*r* = 0.94). Only two studies in Tables 1 and 2 examined time trends. Serum PFOS concentrations increased 3-fold over a 25-year time period in Miyaga, Japan (Harada et al. 2004). Median PFOS concentrations increased approximately 25% between 1974 (median, 25 ng/mL) and 1989 (median, 33 ng/mL) for 58 individuals living in the vicinity of Hagerstown, Maryland (Olsen et al. 2005b). However, only a 9% increase in median PFOS concentrations occurred in two nonpaired populations (*n* = 120 each) residing in the same area and time period. PFOS concentrations did not appear to increase between 1989 and 2001 for this region (Olsen et al. 2003b, 2005b). Individual samples from three large data sets with different age groups predominate the United States findings, as reported in Table 1 (Olsen et al. 2003b, 2004b, 2004c). Sera from children (age 2–12 years, *n* = 598) in 23 states, adult blood donors (age 20–69 years, *n* = 645) from six municipalities in the United States, and elderly (age 65–96 years, *n* = 238) Seattle residents were analyzed for PFOS using identical laboratory methods with comparable findings. Geometric means were 38 (95% CI, 36–39), 35 (95% CI, 33–37), and 31 (95% CI, 29–33) ng/mL, respectively.

Although comparable in average PFOS concentrations, a small number of individuals in each studied population had relatively higher levels than the majority of individuals sampled. The factors that would lead to higher serum PFOS concentrations in some individuals are not completely understood. Some factors that may affect serum PFOS concentrations include proximity to sources of manufacture and use, length of residence in these latter areas, potential product exposures, and possible food and environmental sources. Analysis of 54 pooled serum samples collected from 1,832 participants ≥ 12 years of age in the National Health and Nutrition Examination Survey (NHANES; Calafat et al. 2006) collected in 2001–2002 suggested possible concentration differences by sex (males higher) and ethnicity, in that non-Hispanic whites had statistically significantly higher concentrations compared with non-Hispanic blacks and Mexican Americans (Calafat et al. 2006). An association of higher PFOS serum concentrations with higher fish consumption has been noted in humans (Falandysz et al. 2006) as well as wildlife (Giesy and Kannan 2001). It is notable that PFOS serum concentrations did not strongly correlate with serum concentrations of metabolites of *N*-alkyl-perfluorooctanesulfonamide molecules (Xu et al. 2004) known to be present in products as manufacturing residuals or from degradation of products (Olsen et al. 2003b, 2004b, 2004c). This lack of correlation could be due to differences in the pharmacokinetic behavior of *N*-alkyl-perfluorooctanesulfonamides compared with PFOS or suggest that product exposures may not be a primary source of PFOS in the body.

PFOS data sets have been published for other countries; however, few individual samples have been analyzed. Most of these data were reported by Kannan et al. (2004). In general, the majority of these PFOS concentrations were less than those reported for the U.S. general populations displayed in Table 1. The highest mean PFOS concentration reported by Kannan et al. was found in samples collected from Poland [male, 55 ng/mL (*n* = 10); female, 33 ng/mL (*n* = 15)]. Lower mean PFOS concentrations were reported by Kannan et al. for Korea [male, 27 ng/mL (*n* = 25); female, 15 ng/mL (*n* = 25)]; Belgium [male, 18 ng/mL (*n* = 16); female, 11 ng/mL (*n* = 4)]; Malaysia [male, 13 ng/mL (*n* = 16); female, 12 ng/mL (*n* = 7)]; Brazil [male, 14 ng/mL (*n* = 10); female, 11 ng/mL (*n* = 17)]; Italy [male, 4 ng/mL (*n* = 42); female, 4 ng/mL (*n* = 8)]; Colombia [male, 8 ng/mL (*n* = 31); female, 8 ng/mL (*n* = 25)]; and India [male, 3 ng/mL (*n* = 34); female, 3 ng/mL (*n* = 11)]. In a pilot study, Kubwabo et al. (2004) reported mean concentrations of 30 and 28 ng/mL in 21 and 35 female and male Canadians, respectively. Kärrman et al. (2006) reported geometric mean concentrations of 17 and 16 ng/mL in 40 men and 26 women, respectively, from Sweden. Guruge et al. (2005) reported a mean concentration of 5 ng/mL in 30 adults from Sri Lanka and 0.1 ng/mL in seminal plasma, with a correlation of 0.6 for PFOS between the two matrices. Yeung et al. (2006) reported a mean concentration of 52.76 ng/mL from 85 Chinese samples with considerable differences between nine cities (ranges of means between 3.7 and 79.2 ng/mL). The higher mean PFOS serum concentration was attributed to proximity to industrial production. Other findings reported by 3M (2003) included several blood bank pooled serum samples obtained in 1998 from Belgium, the Netherlands, and Germany that had mean (range in parentheses) serum PFOS concentrations of 17 (5–22) ng/mL, 53 (39–61) ng/mL, and 37 (32–46) ng/mL, respectively.

PFOS serum levels have been measured in 3M employees involved in both the manufacturing of perfluorochemicals and the processing of these compounds into products. Beginning in the late 1970s, measurements were for total organofluorine (Ubel et al. 1980). Since the mid-1990s, measurement of serum PFOS concentrations have been performed as part of employee medical surveillance examinations at the 3M manufacturing facilities in Decatur, Alabama, and Antwerp, Belgium (Olsen et al. 1999, 2003a). Between 1994 and 2000, mean PFOS concentrations approximated 1.0–2.5 μg/mL (1,000–2,500 ng/mL) and ranged between < 0.1 and 12.8 μg/mL, depending upon the job classification. Because employee participation is voluntary, biomonitoring data from these medical surveillance programs may not have provided an adequate characterization of the distribution of serum PFOS concentrations because of possible non-response bias (Olsen et al. 2003d). A study that randomly sampled the Decatur worker population (Olsen et al. 2003d) indicated that serum PFOS concentrations measured during the course of medical surveillance examinations likely presented an unbiased analysis of the overall serum PFOS distribution of the workers. This study showed that mean PFOS serum concentrations for cell and chemical operators were 2.90 μg/mL (geometric mean = 1.97 μg/mL; range, 0.33–6.84 μg/mL) and 1.78 μg/mL (geometric mean = 1.48 μg/mL; range, 0.47–7.26), respectively. These values are approximately 50 times higher than mean (arithmetic or geometric) concentrations measured in the U.S. general population.

Because multiple sources and routes of exposure were probable, estimating external worker PFOS exposure is problematic. Employees may have been exposed to POSF and/or other perfluorochemicals in the manufacturing environment by one or more routes. The primary route of exposure may have varied among employees and depended on several factors, including process conditions, job tasks, work location, personal hygiene, personal habits, and general work practices. Biomonitoring allows for the assessment from all routes of exposure.

## Toxicity and Human Health Data

The toxicological profile of PFOS has been studied extensively (3M Company 2003; Lau et al. 2004; Organisation for Economic Cooperation and Development 2002). Several repeat-dose toxicology studies with PFOS in rodents and nonhuman primates have indicated the potential to reduce body weight and bodyweight gain, increase liver weight, and reduce serum cholesterol. The dose–response curve for mortality in repeat-dose studies is very steep for sexually mature rats and primates (Goldenthal et al. 1978a, 1978b; Seacat et al. 2002b) as well as neonatal rats and mice exposed *in utero* (Lau et al. 2003, Luebker et al. 2005b). Microscopic changes attributable to PFOS include hepatocellular hypertrophy and vacuolation in rats (Seacat et al. 2003) and monkeys (Seacat et al. 2002b), and hepatic necrosis at lethal doses in rats (Goldenthal et al. 1978a). PFOS has been tested for genotoxic activity in a battery of microbial and mammalian systems that included assays for induction of gene mutation (*Salmonella typhimurium* and *Escherichia coli*), gene conversion (*Saccharomyces cerevisiae* D4), chromosomal aberrations (human lymphocytes and mouse bone-marrow micronuclei), and unscheduled DNA synthesis (primary rat hepatocytes) (3M Company 2003). PFOS did not show genotoxic activity in any of these assay systems. In a 2-year dietary study of PFOS in Sprague-Dawley rats (3M Company 2002; Seacat et al. 2002a), hepatocellular adenoma was increased in the high-dose (20 μg potassium PFOS/g feed) males and females. Thyroid follicular cell adenomas were observed in male rats in the high-dose, stop-dose group for which PFOS was eliminated from the diet after 1 year. Decreased serum estradiol was noted in a 6-month monkey study at treatment levels that had a corresponding reduction in serum total cholesterol (Seacat et al. 2002b). Alterations in serum thyroid hormones have been reported (Lau et al. 2003; Luebker et al. 2005b; Seacat et al. 2002b; Thibodeaux et al. 2003) and may be due in part to negative bias in analog measurements and competition between PFOS and thyroid hormones for binding in serum (Tanaka et al. 2005).

In a two-generation reproduction study in rats, mating and fertility were not affected; however, neonatal survival, pup birth weight, and growth of pups in lactation were decreased, and developmental delays were noted (Luebker et al. 2005a). The no observed adverse effect level for these effects was 0.1 mg/kg/day. Reduced postnatal survival and body weight gains may result from *in utero* exposure (Grasty et al. 2003; Lau et al. 2003; Luebker et al. 2005a, 2005b; Thibodeaux et al. 2003). Prenatal developmental toxicity studies of PFOS have been conducted in rats, mice, and rabbits (Case et al. 2001; Lau et al. 2004; Thibodeaux et al. 2003). Prenatal effects in rats administered PFOS during gestation included statistically significant decreases in fetal body weight and statistically significant increases in external and visceral anomalies, delayed ossification, and skeletal variations (Case et al. 2001; Thibodeaux et al. 2003). Maternal toxicity in rats exposed to PFOS during gestation included clinical signs of toxicity and reduction in body weight and food consumption. In rabbits administered PFOS during gestation, statistically significant reductions in fetal body weight and statistically significant increases in delayed ossification were observed; signs of maternal toxicity consisted of abortions and reductions in body weights and food consumption (Case et al. 2001). On the whole, the prenatal developmental effects noted in these studies are consistent between studies, and their significance is somewhat mitigated by the fact that they occur in the presence of maternal deficits in weight gain and feed consumption.

A number of studies have been conducted to investigate the possible modes of action of PFOS. Induction of peroxisome proliferation and associated peroxisomal enzymes (Berthiaume and Wallace 2002; Ikeda et al. 1987; Sohlenius et al. 1993), activation of nuclear receptors (Shipley et al. 2004), interference in lipid metabolism and decreases in serum cholesterol (Haughom and Spydevold 1992; Luebker et al. 2002a, 2002b), interference in mitochondrial bioenergetics (Berthiaume and Wallace 2002; Starkov and Wallace 2002), delays in lung maturation (Grasty et al. 2003), inhibition in gap junctional intercellular communication processes (Hu et al. 2002, 2003), alterations in calcium channels (Harada et al. 2005b), and alterations in thyroid hormone homeostasis (Lau et al. 2003; Luebker et al. 2005b; Tanaka et al. 2005; Thibodeaux et al. 2003) have all been investigated as possible modes of action; however, at present, the mechanisms of action related to the toxicity of PFOS are still not clearly understood. Gene array studies have also been conducted in the rat to investigate changes in mRNA transcriptional responses to PFOS treatment (Hu et al. 2005a, 2005b). Gene expression studies have demonstrated up-regulation of transcription products primarily related to fatty acid metabolism, e.g., β-oxidation pathways, as well as cytochromes P450 and some hormonal regulatory gene transcripts.

In addition to toxicologic studies, epidemiological and medical surveillance studies of exposed 3M Company fluorochemical workers have been conducted by 3M Company for over 25 years. This set of POSF production workers (with potential exposure to PFOS) is the only group reported on from a human health perspective. Clinical tests in medical surveillance examinations in workers have not shown consistent patterns of associations between PFOS serum levels and hematology, hormonal, and clinical chemistry parameters (Olsen et al. 1999, 2003a). A cohort mortality study of the 3M Decatur (Alabama) manufacturing facility showed no statistically significant excess mortality for most types of cancer and for nonmalignant causes (Alexander et al. 2003). However, bladder cancer mortality was elevated (three observed vs. 0.2 expected; standardized mortality ratio 16.12; 95% CI, 3.32–47.14) among male workers who had worked in high PFOS exposure jobs for a minimum of 1 year. It was unclear whether PFOS or other fluorochemicals contributed to the excess of bladder cancer deaths. To further investigate this association, Alexander (2004) mailed a questionnaire to all living members of the original cohort with validation of reported bladder cancers through medical record review when permitted by the subject. A total of 11 cases of primary bladder cancer were identified for the cohort compared with 8.6 expected (standardized incidence ratio 1.28; 95% CI, 0.64–2.29) based on U.S. National Cancer Institute incidence rates. Analyses by duration worked showed no definitive trend.

Worker insurance claims data categorized as episodes of care have also been evaluated (Olsen et al. 2004a). For *a priori* interests, the observed to expected episodes of care experience were comparable for the Decatur fluorochemical and a neighboring film plant (control) employee population for liver tumors, bladder cancer, thyroid and lipid metabolism disorders, and reproductive, pregnancy, and perinatal disorders, and higher for biliary tract disorders and cystitis recurrence. Non-*a priori* associations among the fluorochemical plant workers included benign colon polyps, malignant colorectal tumors, and malignant melanoma. Research is currently being conducted to further investigate these associations.

## Environmental Public Health Use of Biomonitoring Data

Biomonitoring data can be used to examine regional differences and time trends. PFOS concentrations in Charlotte, North Carolina, were the highest of the geographical regions investigated by 3M (Olsen et al. 2003b), and a preliminary screening study conducted by Centers for Disease Control and Prevention to validate methodology showed slightly higher values in Atlanta, Georgia (Kuklenyik et al. 2004). Of six North American areas tested, Stock et al. (2004) reported the largest mean concentration in the troposphere of *N*-methyl perfluorooctanesulfonamidoethanol (359 pg/m^3^) was from the Griffin, Georgia, location. Although the southeastern United States is an area of high carpet and fabric production, it was not possible to establish that this was the reason for the somewhat higher values measured. In addition few data are available that can describe exposure trends. It is assumed that with the voluntary discontinuation of manufacturing of PFOS and POSF-based chemicals by 3M Company, PFOS exposures will eventually diminish.

## Discussion

A review of the data for PFOS related to the exposure, dose, and potential health outcomes parts of the public health paradigm suggest that the PFOS biomonitoring data are relevant for use in public health. Pharmacokinetic data are available to relate serum PFOS concentrations to toxicity and, therefore, to potential health outcomes that may occur across the spectrum of development from *in utero* through adulthood as depicted in the Public Health Paradigm. Analytical methods are sufficiently sensitive, precise, and accurate for the measurement of general-population serum PFOS as a biomarker of exposure. The database for potential health effects is reasonably robust and includes most toxicologic end points as well as several studies of individuals occupationally exposed to PFOS.

The biomonitoring and toxicology data related to PFOS were presented at the International Biomonitoring Workshop on 21–22 September 2004 at Research Triangle Park, North Carolina. Based on the outcomes of that workshop (Albertini et al. 2006) and a general review of the data available for PFOS, the following types of information are considered to be of additional value in understanding the distribution of PFOS in general populations.

- Strengthen the database to allow conversion of whole blood and plasma PFOS concentration values to serum PFOS concentration. Various reports give whole blood measurements converted to estimate serum concentrations by making the assumption that all PFOS is in serum.
- Strengthen the relationship between serum and liver concentrations of PFOS. Although this information is available from toxicology studies, the human data are very limited.
- Bank samples of blood, plasma, or serum for future needs, including early screening investigations (e.g., NHANES).
- Obtain additional data for children, especially children < 2 years of age [e.g., National Children’s Study (2006)].
- Obtain matched serum and urine samples from humans and research animals, where possible (e.g., NHANES), to better understand toxicokinetics.
- Obtain more placental, cord blood, and milk samples [e.g., National Children’s Study (2006)].
- Confirm potential ethnic and sex differences in distributions of blood PFOS concentrations (Calafat et al. 2006).
- Biomonitor additional populations with specific potential occupational, dietary, or consumer exposures to PFOS and related materials; e.g., high consumers of fish (Falandysz et al. 2006), workers with downstream exposure to POSF-based products, and consumers of POSF-based products.
- Build a physiologically based pharmacokinetic (PBPK) model for human handling of PFOS that could explain uptake, clearance, and distribution and exposure pathways by reverse PBPK modeling.

## Correction

This article has been modified from the original manuscript published online. In the section “Exposure Assessment,” the number of pooled samples has been changed from 50 to 54, and the number of participants ≥ 12 years of age has been changed from 1,836 to 1,832. In Table 1, column 5, line 23, “13 (pooled)” has been changed to “14 (pooled).”

## Figures and Tables

**Table 1:** PFOS concentrations (ng/mL) measured in general populations in the United States; samples collected 1974–2003.

Location	Demographic	Type of sample	Year of collection	No.	Average	Range	Reference
Atlanta, GA	Adult females	Serum	2003	10	54^a^	4–164	Kuklenyik et al. 2004
	Adult females	Breast milk	2003	2	< 1	< 1	
	Adult males	Serum	2003	10	58^a^	20–94	
Boston, MA	Adult blood donors	Serum	2001	109	28^b^	< 4–87	Olsen et al. 2003b
Charlotte, NC	Adult blood donors	Serum	2001	96	52^b^	19–166	Olsen et al. 2003b
Hagerstown, MD	Adults	Serum	1974	58^d^	25^c^	(21–34)^e^	Olsen et al. 2005b
	Adults	Serum	1974	120^f^	33^c^	(22–45)^e^	
	Adults	Plasma	1989	58^d^	32^c^	(25–41)^e^	
	Adults	Plasma	1989	120^f^	36^c^	(25–46)^e^	
	Adult blood donors	Serum	2001	108	35^c^	8–226	Olsen et al. 2003b
Los Angeles, CA	Adult blood donors	Serum	2001	125	40^c^	7–205	Olsen et al. 2003b
Portland, OR	Adult blood donors	Serum	2001	107	27^c^	6–1,656	Olsen et al. 2003b
Minneapolis–St. Paul, MN	Adult blood donors	Serum	2001	100	33^c^	8–207	Olsen et al. 2003b
Murray, KY	Adult females	Whole blood	2002	11	66^a^	11–130	Kannan et al. 2004
	Adult males	Whole blood	2002	19	73^a^	19–164	
New York City, NY	Adults	Plasma	2002	79	43^a^	16–83	Kannan et al. 2004
Seattle, WA	Elderly females	Serum	2001	120	32^b^	10–175	Olsen et al. 2004c
	Elderly males	Serum	2001	118	30^b^	< 4–161	
Michigan (central)	Adult females	Serum	2000	46	33^a^	< 2–92	Kannan et al. 2004
	Adult males	Serum	2000	29	33^a^	< 2–124	
23 states	Girls (age 2–12 years)	Serum	1994–1995	298	35^b^	7–165	Olsen et al. 2004b
	Boys (age 2–12 years)	Serum	1994–1995	300	40^b^	11–515	
United States	NHWF	Serum	2001–2002	14 (pooled)	24^a^	NR	Calafat et al. 2006
	NHBF	Serum	2001–2002	6 (pooled)	18^a^	NR	
	MAF	Serum	2001–2002	8 (pooled)	10^a^	NR	
	NHWM	Serum	2001–2002	13 (pooled)	40^a^	NR	
	NHBM	Serum	2001–2002	6 (pooled)	18^a^	NR	
	MAM	Serum	2001–2002	7 (pooled)	14^a^	NR	
Unknown	Adults	Serum	1998	65	28^a^	7–82	Hansen et al. 2001
Unknown	Adult females	Serum	2000	11	17^a^	< 6–32	Olsen et al. 2003c
	Adult females	Liver	2000	14	18^a^	< 7–43	
	Adult males	Serum	2000	13	18^a^	< 7–25	
	Adult males	Liver	2000	16	19^a^	< 5–57	

Abbreviations: MAF, Mexican-American female; MAM, Mexican-American male; NHBF, non-Hispanic black female; NHBM, non-Hispanic black male; NHWF, non-Hispanic white female; NHWM, non-Hispanic white male.

a Mean.

b Geometric mean.

c Median.

d Paired samples (1974 and 1989) from area in vicinity to Hagerstown, Maryland.

e Interquartile range of Hagerstown, Maryland, data.

f Nonpaired samples (1974 and 1989), from area in vicinity of Hagerstown, Maryland.

**Table 2:** PFOS concentrations (ng/mL) measured in general populations in Japan; samples collected 1977–2004.

Location	Demographic	Type of sample	Year of collection	No.	Average	Range	Reference
Akita	Adult females	Serum	1991	40	8^b^	NR	Harada et al. 2004
	Adult females	Serum	1996	60	7^b^	NR	
	Adult females	Serum	2003	66	8^b^	NR	
	Adult males	Serum	2003	50	13^b^	NR	
Hokkaido	Adult females	Whole blood	2003	15	9^a^	5–18	Inoue et al. 2004a
	Fetus (cord blood)	Whole blood	2003	15	3^a^	2–5	
Kyoto	Adult females	Serum	2003	20	14^b^	NR	Harada et al. 2004
	Adult males	Serum	2003	14	28^b^	NR	
	Young adult females	Serum	2004	5	11^a^	9–16	Harada et al. 2005a
	Elderly females	Serum	2004	5	24^a^	16–33	
	Young adult males	Serum	2004	5	13^a^	9–19	
	Elderly males	Serum	2004	5	26^a^	11–49	
Miyagi	Adult females	Serum	1977	39	1^b^	NR	Harada et al. 2004
	Adult females	Serum	2003	23	4^b^	NR	
	Adult males	Serum	2003	32	6^b^	NR	
Tokyo Bay area	Adult females	Whole blood	2002	3	11^a^	5–14	Taniyasu et al. 2003
	Adult females	Whole blood	2002	5	6^a^	2–9	
	Adult females	Serum	2002	3	27^a^	19–41	
	Adult males	Whole blood	2002	2	10^a^	9–11	
Tsukuba and Yokohama	Adult females	Serum	2002	13	18^a^	6–40	Kannan et al. 2004
	Adult males	Serum	2002	25	14^a^	4–38	
Yokohama	Adults	Whole blood	2001	26	8^a^	2–20	Masunaga et al. 2002
Unknown	Adult females	Plasma	2002	10	15^a^	10–19	Inoue et al. 2004b
	Adult males	Plasma	2002	11	19^a^	12–32	

NR, not reported.

a Mean.

b Geometric mean.
